# A Case of Post-Mortem Cesarian Section1Read before the Buffalo Academy of Medicine, Section on Obstetrics.

**Published:** 1898-02

**Authors:** Francis E. Fronczak

**Affiliations:** Visiting physician and surgeon to the Felician Sisters’ Institute, Polish Orphan Asylum and Home for Old and Disabled at Cheektowaga, N. Y.


### Clinical Report.

### A CASE OF POST-MORTEM CESARIAN SECTION.[1]

By FRANCIS E. FRONCZAK, A. M., M. D.,
Visiting physician and surgeon to the Felician Sisters' Institute, Polish Orphan Asylum and Home for Old and Disabled at Cheektowaga, N. Y.

IT DOES not often fall to the lot of a physician of but few months standing to be called upon to perform a Cesarian section delivery, this method being one of the rarest in obstetrics. I trust, therefore, it will be of sufficient value to interest the members of the Academy.

On November 13, 1897, at three o'clock A. M., I was summoned to to attend, according to the statement of the messenger, a case of unconsciousness in child-birth.

The patient, Mrs. A. K., 31 years of age, mother of seven children, all delivered comparatively easily, with no evil after-effects, was lying crosswise in bed. She was cyanosed, breathing stertorously, with eyes unequally dilated. I noticed that only the upper half of her chest was moving. The midwife informed me that she was called to attend the woman in confinement, but that no labor pains were in evidence at any time. Her pulse could hardly be found and the heart-beat was irregular and weak. I at once made an injection of strychnine and later of atropine.

Enquiring into the history, I found the patient expected to be delivered in about six to seven weeks, that she became ill the day before about mid-day, suffering from headache and general malaise; at six in the evening she became worse and went to bed, at eleven she complained of pain in her abdomen, of inability to breathe, of a sensation as of choking, and that her left arm was very lame, and she was afraid she was dying all this time. At midnight she became unconscious and began to breathe in a loud, snoring-like manner. A midwife was sent for; when she arrived she pronounced it to be a case of labor without the labor pains, or a condition something like it, and she advised to watch the patient. Her attendants did watch her until she had a convulsion and her limbs began to turn cold, whereupon I was summoned. I was also informed that the patient was washing all the day before and had also fallen down stairs, striking on her head.

I now examined the woman thoroughly; her pulse was a little stronger, her heart beat sixty times per minute, but she continued to breathe only with the upper half of her chest, the diaphragm being perfectly quiet. No labor pains were noticed, the os was closed, the fetal

1. Read before the Buffalo Academy of Medicine, Section on Obstetrics.

movements could be seen occasionally, and the fetal heart-beat was 124. I at once ordered hot applications to her extremities, and hot bricks placed in the bed. Though the woman had a movement of the bowels early in the evening, I began to make preparations for a lavage of the lower bowel. The heart respiration failing, I injected glonoin and more atropine and morphine. The pulse improved only for a very short time. The respiration being now very shallow, I saw that the patient would not last very long and explained to the relatives that her condition was extreme, that the child in the uterus was alive, and requested to be permitted to deliver it per abdomen in case the mother should die, which was expected almost every moment. They flatly refused. Again and again I injected glonoin and atropine to support the constantly failing heart and respiration. It was all in vain, however, the patient sank rapidly, and died about an hour and a half after my arrival. I listened to the fetal heart sounds: they could be heard but feebly. Again I asked permission to deliver the child by abdominal section, and again I was refused.

I started to go away, when some one of the family thought it would be well for the child to deliver it in the manner proposed, if for no other purpose than for the performance of the baptismal rite. I was called back when but a few feet from the house and told to proceed. I listened to the fetal heart again and I could hear it plainly. Quickly I spread a yard or two of cotton, seized a scalpel, and made an incision, beginning about an inch below the umbilicus, and continuing for about four to five inches downward toward the pubes. There was about a quarter of an inch or more of subcutaneous adipose tissue to be cut through, and the abdominal muscles at their junction at the linea alba. When the uterus was exposed, the fetal movements could be noticed. I opened the uterus with one long incision. But little blood appeared, followed by a gush of amniotic fluid. I pulled the child from the uterus; it breathed but faintly, and its limbs made a convulsive movement or two. I wrapped it quickly in the cotton, tied the cord and cut it. I saw, however, that the child's face was cyanosed and that I would have to resort at once to artificial respiration. I did so, using Schultze's method, and also the method described lately in *Pediatrics*, in which the child is laid on its back, the operator putting one hand on the chest and taking hold of the legs with the other, alternately raises them over on the abdomen and presses the chest, thus bringing about inspiratory and expiratory movements. I labored hard and earnestly, but the child lived only a very short time. It was a female, fully developed, weighing about seven pounds, and in the uterus it was a vertex presentation.

The question may now be asked, to what was the mother's death due? It might have been angina pectoris, it might have been some injury sustained by the fall, this injury interfering with the movements of the diaphragm. Finally, eclampsia also claims our attention. Each of the above-mentioned conditions had its characteristic symptoms, each in itself a sufficient cause of fatality. The child's death no doubt can be attributed to asphyxia, due to carbonic acid gas generated in the mother's system, as the respiration was not sufficient to fully oxidise the maternal blood. Finally, was I justified in performing Cesarean section ? I think it was my duty to perform this operation in view of the child's signs of life. It is, I believe, the conviction among the medical profession and even among the educated laity, that to deliver a possibly living child from its dead mother's uterus is justifiable. In Austria, I understand, there exists a law that a child should be delivered through abdominal section at its mother's death.

508 FILLMORE AVENUE.

